# Familial Retinoblastoma: Raised Awareness Improves Early Diagnosis and Outcome

**DOI:** 10.1155/2017/5053961

**Published:** 2017-03-02

**Authors:** Ibrahim Al-Nawaiseh, Aseel Q. Ghanem, Yacoub A. Yousef

**Affiliations:** Department of Surgery, King Hussein Cancer Center, Amman, Jordan

## Abstract

*Purpose*. To study the impact of awareness of retinoblastoma in the affected families on the management and outcome of familial retinoblastoma patients. *Methods and Materials*. This is a retrospective, clinical case series of 44 patients with familial retinoblastoma. Collected data included patient's demographics, laterality, family history, age at diagnosis, presenting signs, treatment modalities, tumor stage, eye salvage rate, metastasis, and mortality. *Results*. Out of 200 retinoblastoma patients in our registry, 44 (22%) patients were familial, 18 were probands, and 26 were second, third, or fourth affected family members. There were 76 affected eyes: 31 eyes of probands and 45 eyes of the other affected family members. Among probands, all patients (100%) had at least one eye enucleated: 58% (18 eyes) of the affected eyes were enucleated and 32% (10 eyes) of the affected eyes were radiated. On the other hand, among the nonprobands, only 20% had one eye enucleated, and only 4 eyes (9%) received radiation. The eye salvage rate was significantly higher in the nonprobands than in the probands in this series (*p* = 0.00206). Patients diagnosed by screening (38%) had excellent visual outcome, and both eyes were salvaged. *Conclusion*. Awareness of families of the possibility of retinoblastoma and adequate screening led to a significantly higher rate of eye salvage in patients with familial retinoblastoma.

## 1. Introduction

Retinoblastoma (Rb) is the most common childhood intraocular tumor, and 90% of the cases are diagnosed before the age of 5 years worldwide [1, 2]. Globally, the incidence is approximately 1 in 15,000 to 20,000 live births [3, 4]. An earlier epidemiological study from Jordan has reported an incidence of 9.32 cases per million children among 0–5 years of age [4]. Approximately 20% of cases reported from Jordan are familial whereas only 10% of cases are familial in western countries [4, 5]. The ratio of male : female was 2.3 : 1, and the number of bilateral cases was 47.5%, which is higher than the bilateral Rb cases reported from western countries [4]. This might be because of the large-sized families in Jordan. The 2012 population census of Jordan has reported an average family household size of 5.1, thus increasing the incidence of familial Rb [6].

Over the years, the prognosis of Rb has improved because of better treatment modalities and early diagnosis. One of the most important poor prognostic factors for the management of Rb is the delay in diagnosis [7, 8]. As patients in the developing countries are usually diagnosed in late stages of the disease, because of the difficulty in getting adequate health care, they present with a more advanced stage and, therefore, have less survival rates. The patients in the developed countries and those who have better socioeconomic status generally present at an earlier stage and get adequate health care leading to a higher survival rate as compared to patients from developing countries [7, 8].

Due to the rarity of the disease, people are generally not aware of the possibility of eye cancer [3]. The diagnosis of Rb in the first child (proband) is often delayed, but once the family becomes aware of the risk of developing Rb in the other newborns (nonprobands), they may consider any abnormal sign more seriously and, even more, they may get the nonprobands screened for earlier diagnosis and better outcome. We assume that the parental awareness may help in early diagnosis and better outcome among the nonprobands. Soliman et al., while conducting a study in Egypt, had proposed a similar hypothesis and has shown that awareness increased among parents with a proband child as compared to those without proband and further improved the outcome [9].

We aimed to evaluate the characteristics and management outcomes for the familial Rb cases in Jordan and the impact of awareness of these families on the detection and outcome of Rb in nonprobands.

## 2. Materials and Methods

This was a retrospective case series of 76 eyes of 44 consecutive familial Rb patients who had been managed in a single tertiary cancer centre in Jordan (KHCC). Patients enrolled between March 2008 and March 2016 were analyzed. Data was collected from the medical records and Ret-Cam images taken along with. Inclusion eligibility criterion included clinical and/or pathological diagnosis of Rb with a family history of Rb. Thus, Rb patients without a family history of Rb were excluded from this study. The first family member with a diagnosis of Rb was called a proband, and other affected family members were called nonprobands. Information regarding the following parameters was collected: patients' age at diagnosis, gender, laterality, affected site, Reese-Ellsworth (RE) group at diagnosis, International Classification of Rb stage at diagnosis (Table 1), presenting signs and symptoms, management, eye salvage, visual outcome, metastasis, any other malignancy, and mortality. Selection and data collection required access to patients' medical records, Ret-Cam images, and medical reports for patients who were diagnosed and/or managed initially outside our centre. This study was approved by the institutional review board.

### 2.1. Treatment Methods

We used a combination chemotherapy regimen of CVE (carboplatin, vincristine, and etoposide). Each CVE cycle was repeated every 4 weeks for a total of 6 to 8 cycles according to patient's condition and tumor status. Ocular oncology follow-up was provided with examination under anesthesia before each and every cycle of chemotherapy and every 4 weeks thereafter. Fundus photos were taken using a Ret-Cam II (Clarity Medical System, Pleasanton, CA, USA). Combination focal therapy was applied as needed as transpupillary thermotherapy (TTT) and triple freeze thaw cryotherapy (MIRA CR 4000). External beam radiation therapy was administered when needed in a consistent fashion by applying 45 Gy in 25 fractions.

### 2.2. Statistical Analysis

Descriptive analysis was carried out using mean, median, and range. Comparative analysis was carried out between probands and nonprobands, and *p* value was measured using Fisher's exact test for analyzing the predictive power of each factor.

## 3. Results

Out of the 200 retinoblastoma patients (between March 2008 and March 2016) admitted in the hospital, 44 (22%) patients had familial Rb, 18 were probands, and 26 were second, third, or fourth affected family member (nonprobands). There were 76 affected eyes: 31 eyes for probands and 45 eyes for the other affected family members.

### 3.1. Proband Group

Out of the 31 affected eyes, 20 (64%) were right eyes. The mean age at diagnosis was 17 months. Out of the 18 patients, 13 (72%) were males and 5 (28%) were females. Thirteen (72%) patients had bilateral Rb, and 5 (27%) had unilateral Rb. Leukocoria was the most common presenting sign in 16 (89%) patients, followed by strabismus in 2 (11%) cases. All the affected eyes had multifocal disease. At the time of diagnosis, 26 (84%) eyes were classified into group D or E and 5 (16%) eyes were placed into group A, B, or C [1].

Treatment and outcome: systemic chemotherapy was used in the management of 12 (67%) patients. Focal therapy (TTT or cryotherapy) was applied in 11 (35%) eyes, and 10 (32%) eyes received external beam radiotherapy. Fourteen (45%) eyes in this group were salvaged by the last date of follow-up while enucleation was mandatory for 17 (55%) of the treated eyes. In this group, all patients (100%) had enucleation of at least one eye. At the last date of follow-up, best-corrected visual acuity was less than 0.5 in 6 (18%) eyes and better than 0.5 in 8 (26%) eyes (vision assessment was not possible for uncooperative patients 17 (55%) eyes) (Table 2). One patient had secondary malignancy (maxillary osteosarcoma), and no case had metastasis or died by the last date of follow-up.

### 3.2. Nonproband Group

Twenty-six patients (59%) of total cases of familial Rb were nonprobands. There were 45 affected eyes; 24 (53%) of them were in the right eye. There were 11 (42%) males and 15 (58%) females. The mean age at diagnosis was 8 months. Eighteen (73%) patients had bilateral disease, and the rest had unilateral disease.

Leukocoria was the presenting sign among 10 (38%) patients, followed by strabismus among 6 (23%) cases. Ten (38%) patients had been diagnosed by screening. Thirty-nine eyes (87%) had multifocal disease, and 6 eyes (13%) had unifocal disease. Twenty-six eyes (57%) had tumor in stage A or B, and 19 eyes were grouped C, D, or E at the time of diagnosis [1].

Treatment and outcome: systemic chemotherapy was used for the management of 20 patients (77%), while 6 patients (23%) did not receive systemic chemotherapy. Focal therapy (TTT or cryotherapy) was applied to 35 eyes (78%) in this group, and 10 (22%) eyes did not receive focal treatment. Four (8%) patients received external beam radiation. Thirty-six (80%) eyes were salvaged at the last date of follow-up while enucleation was mandatory for 9 (20%) of the treated eyes. Out of the 33 eyes in groups A, B, and C, 32 (97%) eyes were salvaged while out of the 12 eyes in groups D and E, only 4 (33%) eyes were salvaged [1].

At the last date of follow-up, visual acuity was less than 0.5 in 5 (20%) patients and better than 0.5 in 13 (50%) patients (vision assessment was not applicable for 8 (30%) patients) (Table 1). There was no case of secondary malignancy, or metastasis, and no deaths occurred up to the last date of follow-up.

## 4. Discussion

The results of the study are in favor of our hypothesis. We had hypothesized that an early diagnosis can be achieved among familial cases (other than the first affected patient in each family), which may further lead to better outcome. It is assumed that the families who have one child (probands) with retinoblastoma will be more aware after the birth of second or subsequent children. The results show that 58% of the nonproband cases were diagnosed as group A or B [1] (early intraocular stage), whereas only 3% of the probands were in these 2 groups. On the other hand, 84% of the probands were diagnosed in stage D or E (advanced intraocular stage), while only 26% of the nonprobands were diagnosed in these 2 groups. The mean age of diagnosis in the nonproband group was 8 months as compared to 17 months in the proband group, and 10 cases were detected by screening in the nonproband group. The earlier diagnosis among the nonprobands can be attributed to the awareness among the parents and their initiative to undertake screening. A similar retrospective study by Soliman et al. found that at the time of probands, none of the parents without probands knew about screening for Rb among newborns, whereas all parents with probands knew about it at the time of the second child [8]. The results of our study and those by Soliman et al. show that the screening for Rb among *at risk* patients helps in earlier diagnosis of Rb in countries like Jordan and Egypt, respectively [9]. This increasing trend of screening and awareness was significantly less than the developed world a few years back. Chantada et al. while comparing the screening rates among developing countries and the USA found that the screening of Rb was significantly less in developing countries (Argentina, Brazil, Jordan, Turkey, and Venezuela) [10].

The difference in the rate of salvage and other outcome parameters shows the impact of awareness and screening. Among the probands, there was enucleation of at least one eye with an eye salvage rate of 54%, while in nonprobands who were diagnosed earlier, the eye salvage rate was significantly higher at 80%. Retinoblastoma management guidelines recommend that the early diagnosed tumors (group A and few of group B tumors) can be treated with focal therapy even without chemotherapy, while more advanced tumors need chemotherapy and sometimes radiation and/or enucleation [11]. Our results show that in the proband group, 67% of patients received chemotherapy, while the rest did not undergo chemotherapy because of late diagnosis and thus had to undergo enucleation. In contrast to this, 77% of the nonproband cases received chemotherapy and 23% were treated by focal therapy as most of them had presented during the early stage. The enucleation rate was lower in the nonproband group. Soliman et al. have also shown that early eye screening decreased the tumor burden (*p* = 0.03), lowered the treatment burden (*p* = 0.04), and had a higher rate of ocular salvage (*p* = 0.01) and better visual outcome (*p* = 0.01) [9].

Our results have shown a younger mean age (17 and 8 months in the proband and nonproband groups, resp.) at the time of diagnosis as compared to earlier reports, which have shown the mean age to be 24 months [12]. In our study, approximately 80% of the cases were diagnosed before the age of 3 or 4 years. The younger age might be because of the parental awareness. We had earlier reported a median age of 12 months from a retrospective analysis of 10 years from our hospital for the patients enrolled until the year 2013 [13].

The signs and symptoms of Rb depend on its size and location. Leukocoria (which is seen when the tumor is large in size) is the most frequent presenting sign of Rb, found in approximately 50–60% of cases, followed by strabismus (25%) and inflammatory signs (6–10%) [14–17]. In our groups, 83% of probands presented with leukocoria, followed by strabismus (11%); on the other hand, in the nonproband group as 38% were discovered by screening, there were no presenting symptoms, while 38% presented with leukocoria and 23% presented with squint. This difference in presentation again highlights the significance of awareness among the parents.

Early diagnosis and therefore management of retinoblastoma by focal therapy alone in the early stages rather than chemotherapy and possible radiation in the late stages will have an important impact on both social and economic status of the family and the community since focal therapy is much cheaper. In addition to that, early diagnosis means better visual outcome, so the affected patient will not be dependent in the future by keeping their eyes and vision and, therefore, will serve themselves without being an overload on the economy of the country specifically in the poor and developing countries.

Our results demonstrate the importance of early diagnosis of Rb, which is possible through education of the community and training of health professionals. They can thus help in early identification, diagnosis, and initiation of management to improve the outcome. Results show that screening of the *at risk* population can be a diagnostic tool. In our study, patients diagnosed at an earlier stage had better outcome because the families were more aware of the signs of Rb and received medical care at an early stage. The retrospective study design is a limitation of our study. However, the long duration of 8 years as the inclusion period is a major strength of our study.

In conclusion, family awareness of the possibility of retinoblastoma and adequate screening (even in the absence of genetic testing) are associated with earlier diagnosis and higher rates of eye salvage in patients with retinoblastoma.

## Figures and Tables

**Table 1:** International intraocular Rb classification [1].

*Group A: very low risk*
Small discrete tumors not threatening vision
(i) All tumors are 3 mm or smaller, confined to the retina
(ii) Located at least 3 mm from the foveola and 1.5 mm from the optic nerve
(iii) No vitreous or subretinal seeding

*Group B: low risk*
No vitreous or subretinal seeding
(i) Tumors of any size or location not in group A
(ii) No vitreous or subretinal seeding
(iii) Subretinal fluid no more than 5 mm from tumor base

*Group C: moderate risk*
Focal vitreous or subretinal seeding and discrete retinal tumors of any size and location
(i) Local, fine, and limited seeding
(ii) Discrete intraretinal tumors of any size and location
(iii) Up to one quadrant of subretinal fluid

*Group D: high risk*
Diffuse vitreous or subretinal seeding
(i) Diffuse intraocular disseminated disease
(ii) Extensive or “greasy” vitreous seeding
(iii) Subretinal seeding may be plaque-like
(iv) More than one quadrant retinal detachment

*Group E: very high risk*
Very high risk with one or more of the following:
(i) Irreversible neovascular glaucoma
(ii) Massive intraocular hemorrhage
(iii) Aseptic orbital cellulitis
(iv) Tumor anterior to anterior vitreous face
(v) Tumor touching the lens
(vi) Diffuse infiltrating Rb
(vii) Phthisis or prephthisis

**Table 2:** Clinical features and outcome of eye with retinoblastoma in both proband and nonproband groups.

		Proband		Nonproband		*p* value
Presenting symptom	Leukocoria	15	83%	10	38%	0.1
	Squint	2	11%	6	23%	
	Screening	0	0%	10	38%	

IIRC group at diagnosis	A	0	0%	12	27%	0.0001
	B	1	3%	14	30%	
	C	4	13%	7	16%	
	D	20	65%	8	17%	
	E	6	19%	4	9%	

Eye salvage rate^∗∗^	Yes	14	45%	36	80%	
	No	17	55%	9	20%	

BCVA	Less than 0.5	6	18%	5	20%	
	Better than 0.5	8	26%	13	50%	

IIRC: International Intraocular Retinoblastoma Classification; BCVA: best-corrected visual acuity at the last date of follow-up per eye.

^∗∗^Eye salvage rate per eye.
